# Temporal muscle thickness is associated with immunotherapy outcomes in head and neck squamous cell carcinoma

**DOI:** 10.1371/journal.pone.0356013

**Published:** 2026-08-13

**Authors:** Hirotaka Eguchi, Kiyohito Hosokawa, Ryota Kawano, Yukinori Takenaka, Hiroshi Kato, Toshihiro Kishikawa, Masami Suzuki, Motoyuki Suzuki, Takeshi Tsuda, Ryohei Oya, Hidenori Inohara

**Affiliations:** 1 Department of Otorhinolaryngology-Head and Neck Surgery, The University of Osaka Graduate School of Medicine, Suita, Osaka, Japan; 2 Department of Otorhinolaryngology-Head and Neck Surgery, Shiga University of Medical Science, Otsu, Japan; All India Institute of Medical Sciences, INDIA

## Abstract

**Background:**

Reduced skeletal muscle mass may impair outcomes of immune checkpoint inhibitors in recurrent or metastatic head and neck squamous cell carcinoma. We evaluated whether temporal muscle thickness (TMT) on routine head computed tomography was associated with treatment response and survival.

**Methods:**

We retrospectively analyzed 109 patients treated with nivolumab or pembrolizumab between 2017 and 2023 (89 men [81.7%], 20 women [18.3%]; median age 69 years [range 21–88]). TMT was measured on CT images at the level where the Sylvian fissure was most clearly visualized, perpendicular to the temporalis muscle long axis, on both sides, and the mean of the bilateral values was used. Sex-specific cutoffs were defined by time-dependent receiver operating characteristic analysis for 12-month overall survival (5.53 mm for men; 3.92 mm for women). Associations with objective response and survival were assessed using logistic regression and Cox proportional hazards models.

**Results:**

Objective response was evaluable in 105 patients (complete response 12, partial response 30, stable disease 18, progressive disease 45), yielding a response rate of 40.0% and a disease control rate of 57.1%. High temporal muscle thickness was associated with response (univariable odds ratio 2.64, 95% confidence interval 1.18–5.90; p = 0.018) and remained significant after adjustment for chemotherapy (adjusted odds ratio 2.42, 95% confidence interval 1.05–5.58; p = 0.038). One-year progression-free survival was 36.5% in the high group versus 15.8% in the low group (absolute difference 20.7%; p = 0.010; adjusted hazard ratio 0.57, 95% confidence interval 0.36–0.90; p = 0.017). One-year overall survival was 73.1% versus 47.4% (absolute difference 25.7%; p = 0.001; adjusted hazard ratio 0.54, 95% confidence interval 0.34–0.86; p = 0.010).

**Conclusion:**

Temporal muscle thickness, obtainable from routine head computed tomography, was independently associated with response and survival in patients receiving immune checkpoint inhibitors and may serve as a practical host-related imaging marker for prognostic risk stratification.

## Introduction

Head and neck squamous cell carcinoma (HNSCC) arises from the mucosal surface of the upper aerodigestive tract, such as the pharynx and larynx. Due to the affected anatomic site, dysphagia and malnutrition are commonly observed in patients with HNSCC [1]. Surgery often deforms the pharynx and causes stricture. Radiation and chemotherapy cause xerostomia, fibrosis, and edema of the neck. All of these factors exacerbate dysphagia. As a result, recurrent and metastatic (R&M) HNSCC cases are in worse nutritional status than previously untreated cases.

Malnutrition is diagnosed based on the presence of phenotypic and etiologic criteria [2]. One of the phenotypic criteria is reduced muscle mass. Although whole-body muscle mass is quantified using dual-energy X-ray absorptiometry, bioelectrical impedance analysis, or magnetic resonance imaging [3], computed tomography (CT) image at the L3 level has been commonly used in the oncologic field [4]. However, routine images for the follow-up of HNSCC do not include L3 level image. Therefore, several researchers used C3 level images and demonstrated the prognostic utility of muscle mass at C3 [5,6]. HNSCC treatment substantially affects the neck muscles. Surgery sometimes involves excision of the neck skeletal muscle. Radiation therapy can cause neck muscle atrophy. Thus, muscle mass at C3 is not an ideal surrogate for whole-body muscle mass in patients with previously treated HNSCC.

Recently, temporal muscle thickness (TMT) has been shown to correlate with whole-body skeletal muscle mass and is a prognostic marker of brain tumors [7,8]. However, only two reports have been published regarding the prognosis of HNSCC and TMT [9,10]. Moreover, these reports analyzed curative treatment cases, and no report has investigated the association between TMT and immune checkpoint inhibitor (ICI) outcomes in patients with HNSCC.

This study aimed to investigate whether TMT is a prognostic biomarker for R/M HNSCC treated with ICI.

## Methods

### Patients and data extraction

The inclusion criteria were as follows: (1) histologically or cytologically confirmed HNSCC; (2) treatment with nivolumab or pembrolizumab for R/M HNSCC at the Department of Otorhinolaryngology-Head and Neck Surgery, The University of Osaka Hospital, between May 2017 and May 2023; and (3) axial CT images at the level of the Sylvian fissure and orbital roof within three months before the start of ICI treatment. The exclusion criteria were as follows: (1) history of ICI therapy for other malignancies; (2) tumors involving or disfiguring the temporal muscle; and (3) insufficient clinical data. A retrospective chart review was performed on records that met the abovementioned criteria. The following data were collected: sex, age, Eastern Cooperative Oncology Group performance status (PS), TMT within 3 months prior to initiation of ICI therapy, tumor site, human papillomavirus (HPV) status, ICI, and chemotherapy regimens.

### Measurement of TMT on CT images

TMT was measured perpendicular to the long axis of the temporal muscle in images where the Sylvian fissure was most clearly visualized [8]. TMT on the right and left sides was measured, and the mean was used as the TMT of the patient.

### Oncologic outcomes

The objective response was evaluated using the Response Evaluation Criteria in Solid Tumours guidelines, version 1.1 [11]. The response rate (RR) was defined as the percentage of patients who achieved complete response (CR) or partial response (PR). Disease control rate (DCR) was defined as the percentage of patients who achieved CR, PR, or stable disease (SD). Overall survival (OS) was defined as the time from initiation of ICI treatment to death from any cause. Progression-free survival (PFS) was defined as the time from initiation of ICI treatment to disease progression or death from any cause.

### Ethics approval

The study protocol was approved by the Institutional Review Boards of The University of Osaka (16329). The study was conducted in accordance with the 1964 Declaration of Helsinki and its later amendments. Given the retrospective nature of the study, the requirement for written informed consent was waived by the ethics committees, as permitted by the ethical guidelines for medical and health research involving human subjects issued by the Ministry of Health, Labour and Welfare of Japan. Clinical data were accessed for research purposes between December 1, 2024 and March 31, 2025. Although the authors had access to identifiable information during data collection, all data were anonymized prior to analysis.

### Statistical analysis

Associations between categorical variables and those between continuous and categorical variables were assessed using the chi-square and Kruskal-Wallis tests, respectively. The odds ratio (OR) for objective response was calculated using a logistic regression model. Survival was estimated using the Kaplan–Meier method and compared using the log-rank test. The hazard ratio (HR) for survival outcomes was calculated using the Cox proportional hazards model. A probability (*p*) value of <0.05 was considered statistically significant. The sex-specific cutoff value for TMT was determined using time-dependent receiver operating characteristic (ROC) analysis for survival at 12 months. These analyses were performed using JMP version 17 statistical software (SAS Institute Japan, Tokyo, Japan) and R version 4.2.1 (R Foundation for Statistical Computing, Vienna, Austria) and the timeROC and survival packages.

Sensitivity analyses were performed to evaluate the robustness of the association between TMT and clinical outcomes. First, mean TMT was analyzed as a continuous variable in logistic regression and Cox proportional hazards models. Second, BMI was additionally included in the Cox models to assess whether the association between TMT and survival was explained by BMI. Third, treatment-related variables, including chemotherapy use and ICI agent, were examined to assess potential confounding by treatment regimen.

## Results

### Patient characteristics

We identified 130 patients who met the inclusion criteria. We then applied the exclusion criteria (which were not mutually exclusive): non-SCC (n = 18), lack of CT images at the desired level (n = 20), and temporal muscle involvement by the tumor (n = 1). Consequently, 109 patients were included in the analysis. The clinicopathological characteristics of the patients are presented in Table 1. The male-to-female ratio was 89:20, and 66% of patients were aged ≥ 65 years at the start of ICI treatment. The most common primary site was the hypopharynx, followed by the oropharynx and oral cavity. Cytotoxic drug-containing regimens were administered to 33% of the patients.

**Table 1:** Patient characteristics.

	Median(range)	No.	%
Sex			
Male		89	81.7
Female		20	18.3
Age, years	69 (21–88)		
ECOG-PS			
0, 1		94	86.2
2 or more		15	13.8
Primary site			
Nasopharynx		13	11.9
Oropharynx		23	21.1
HPV		5	4.6
non-HPV		18	16.5
Hypopharynx		30	27.5
Oral cavity		14	12.8
Larynx		9	8.3
Other		20	18.3
Regimens			
Nivolumab		59	54.1
Pembrolizumab		50	45.9
with FP		36	33.0
without FP		14	12.9
TMT			
Male	5.35 (2.18–9.13)		
Female	4.06 (2.50–7.77)		
BMI	20.0 (12.6–61.2)		

Abbreviation: BMI, body mass index, ECOG-PS; Eastern Cooperative Oncology Group-Performance Status, HPV; human papillomavirus, FP; fluorouracil and platinum, TMT; temporal muscle thickness.

The TMT ranged from 2.18–9.13. The cutoff values determined using time-dependent ROC analyses were 5.53 for male and 3.92 for female, respectively.

### TMT and other patients’ factors

There was no association between TMT and PS, sex, or primary site (p = 0.407, 0.061, and 0.442, respectively). However, the TMT in males tended to be higher than that in females (median TMT 5.35 and 4.06, respectively, (p = 0.061)). The correlation coefficients between TMT and age, height, body weight, and body mass index were −0.18 (p = 0.059), 0.14 (p = 0.149), 0.06 (p = 0.511), and 0.009 (p = 0.923), respectively, and no significant correlation was found.

Treatment-related variables were not significantly imbalanced between the low- and high-TMT groups. Chemotherapy use did not differ significantly according to TMT group (p = 0.249), and the distribution of ICI agent, nivolumab versus pembrolizumab, was also comparable between groups (p = 0.476). BMI did not differ significantly between the low- and high-TMT groups (Wilcoxon test, p = 0.566).

### Objective response

Of the 109 patients, 105 were evaluated for objective response. Overall, CR, PR, SD, and progressive disease were observed in 12, 30, 18, and 45 patients, respectively. RR and DCR were 40.0% and 57.1%, respectively.

The median TMT values among responders and non-responders were 5.80 and 4.85, respectively (p = 0.061). The median TMT among patients who achieved disease control and who did not were 5.51 and 4.96, respectively (p = 0.368).

The odds ratios for response and disease control according to clinicopathological factors in the logistic regression analyses are shown in Table 2 and 3. Among these factors, TMT and concomitant use of ICI with chemotherapy were significantly associated with response (OR 2.64, 95% CI 1.18–5.90 and 3.50, 95% CI 1.50–8.17, respectively). When adjusted for chemotherapy administration, high TMT was independently associated with better response (OR 2.42, 95% CI 1.05–5.58). On the other hand, chemotherapy was the only prognosticator of disease control (OR 3.61, 95% CI 1.48–9.57) (Table 3).

**Table 2:** Odds ratio for response.

	Univariate			Multivariate		
Variables	OR	(95% CI)	*p* value	OR	(95% CI)	*p* value
Sex						
Male vs female	1.18	(0.42–3.28)	0.756			
Age						
≥ 65 vs < 65	1.54	(0.66–3.57)	0.315			
PS						
≥ 2 vs 0, 1	1.33	(0.41–4.29)	0.629			
HPV						
positive	1.00	(0.16–6.25)	1.000			
Chemotherapy						
Yes vs no	3.50	(1.50–8.17)	0.004	3.26	(1.37–7.76)	0.008
TMT						
High vs low	2.64	(1.18–5.90)	0.018	2.42	(1.05–5.58)	0.038

Abbreviations: CI, confidence interval, HPV, human papillomavirus, OR, odds ratio, TMT, temporal muscle thickness.

**Table 3:** Odds ratio for disease control.

	Univariate			Multivariate		
Variables	OR	(95% CI)	*p* value	OR	(95% CI)	*p* value
Sex						
Male vs female	0.96	(0.35–2.63)	0.942			
Age						
≥ 65 vs < 65	1.31	(0.58–2.95)	0.514			
PS						
≥ 2 vs 0, 1	0.60	(0.19–1.94)	0.396			
HPV						
positive	3.14	(0.34–29.1)	0.314			
Chemotherapy						
Yes vs no	3.78	(1.51–9.48)	0.005	3.61	(1.48–9.57)	0.004
TMT						
High vs low	1.71	(0.78–3.75)	0.177	1.52	(0.67–3.47)	0.313

Abbreviations: CI, confidence interval, HPV, human papillomavirus, OR, odds ratio, PS, performance status, TMT, temporal muscle thickness

### TMT as a prognostic factor for survival

**Fig 1:**
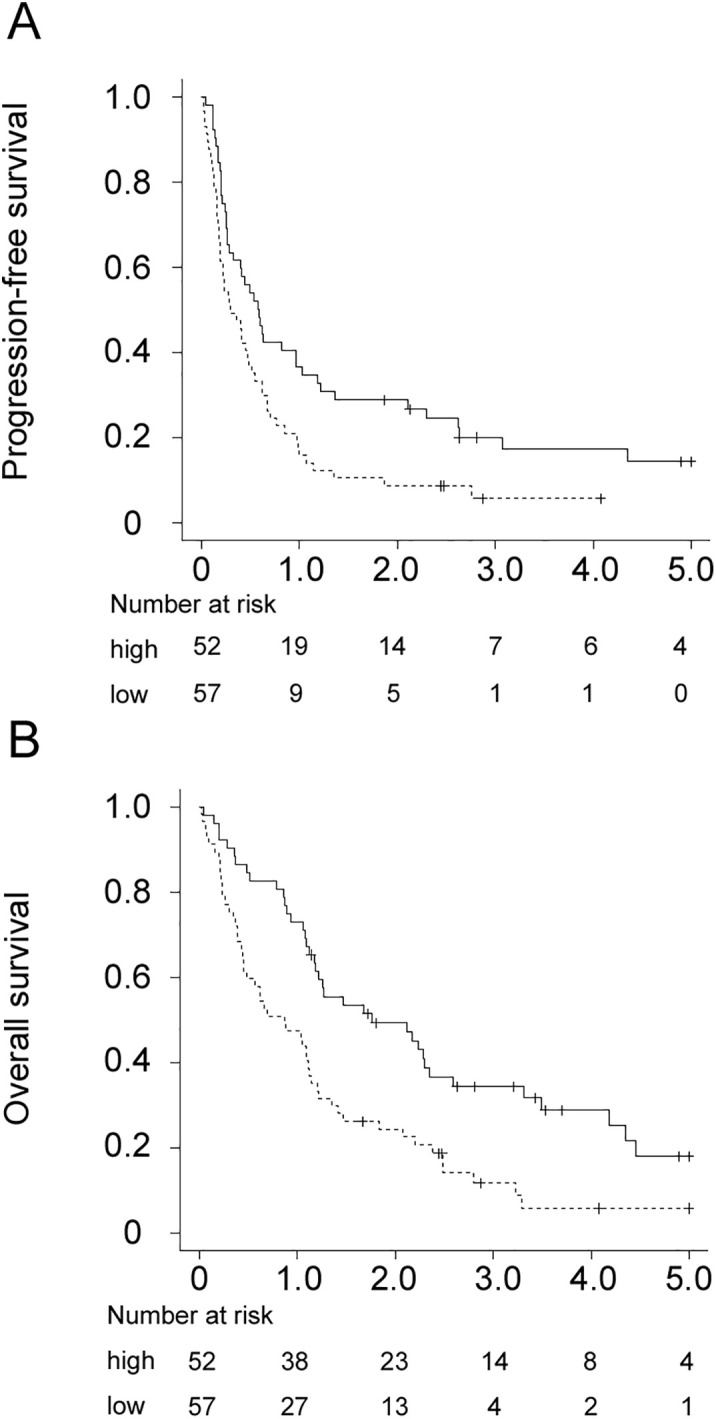
Kaplan–Meier curves for PFS (A) and OS (B) stratified by temporal muscle thickness. Sex-specific cutoffs were used to define high and low groups. Numbers at risk are shown below the plots; tick marks indicate censoring. PFS: HR **0.57** (95% CI **0.36–0.90**), **p** = **0.017**, events **96/109**. OS: HR **0.54** (95% CI **0.34–0.86**), **p** = **0.010**, deaths **89/109**.

Fig 1A shows PFS in the low and high TMT groups. The 1-year PFS rates for low and high TMT groups were 15.8% and 36.5%, respectively (p = 0.010). Table 4 shows univariate analyses for PFS according to clinicopathological variables. Among these variables, only TMT was significantly associated with PFS (Table 4). Multivariate analysis demonstrated that TMT was independently associated with PFS (HR 0.57, 95% CI 0.36–0.90) (Table 4).

**Table 4:** Hazard ratio for progression-free survival.

	Univariate			Multivariate		
Variables	HR	(95% CI)	*p* value	HR	(95% CI)	*p* value
Sex						
Male vs female	1.49	(0.85–2.63)	0.167			
Age						
≥ 65 vs < 65	1.13	(0.73–1.73)	0.587	0.94	(0.59–1.50)	0.769
PS						
≥ 2 vs 0, 1	1.45	(0.82–2.56)	0.201	1.47	(0.83–2.61)	0.189
HPV						
positive	0.72	(0.26–19.7)	0.524	0.95	(0.33–2.69)	0.920
Chemotherapy						
Yes vs no	0.87	(0.57–1.32)	0.507	0.96	(0.62–1.48)	0.853
TMT						
High vs low	0.58	(0.39–0.88)	0.010	0.57	(0.36–0.90)	0.017

Abbreviations: CI, confidence interval, HPV, human papillomavirus, HR, hazard ratio, PS, performance status, TMT, temporal muscle thickness.

Fig 1B shows OS in the low and high TMT groups. The 1-year OS rates for low and high TMT groups were 47.4% and 73.1%, respectively (p = 0.001). In univariate analyses, age, PS, and TMT were associated with OS (Table 5). Multivariate analysis revealed PS and TMT as independent prognostic factors for OS (HR 1.93, 95% CI 1.08–3.44 and HR 0.54, 95% CI 0.34–0.86, respectively) (Table 5).

**Table 5:** Hazard ratio for overall survival.

	Univariate			Multivariate		
Variables	HR	(95% CI)	*p* value	HR	(95% CI)	*p* value
Sex						
Male vs female	1.68	(0.93–3.01)	0.083			
Age						
≥ 65 vs < 65	1.58	(1.00–2.49)	0.048	1.35	(0.83–2.21)	0.232
PS						
≥ 2 vs 0, 1	1.88	(1.06–3.34)	0.031	1.93	(1.08–3.44)	0.026
HPV						
positive	0.61	(0.19–1.94)	0.403	0.74	(0.22–2.43)	0.618
Chemotherapy						
Yes vs no	0.87	(0.56–1.36)	0.542	0.95	(0.61–1.49)	0.823
TMT						
High vs low	0.49	(0.32–0.75)	0.001	0.54	(0.34–0.86)	0.010

Abbreviations: CI, confidence interval, HPV, human papillomavirus, HR, hazard ratio, PS, performance status, TMT, temporal muscle thickness.

Sensitivity analyses treating TMT as a continuous variable supported the association between TMT and survival outcomes. Higher mean TMT was significantly associated with longer PFS after adjustment for age, PS, HPV status, and chemotherapy use (adjusted HR per 1-mm increase, 0.84; 95% CI, 0.72–0.98; p = 0.022), and this association was essentially unchanged after additional adjustment for BMI. Similarly, higher mean TMT was significantly associated with longer OS after adjustment for age, PS, HPV status, and chemotherapy use (adjusted HR per 1-mm increase, 0.84; 95% CI, 0.71–0.98; p = 0.032), and after additional adjustment for BMI (S1 Table).

## Discussion

This study demonstrated that TMT was associated with objective response and survival outcomes in patients with R/M HNSCC treated with ICIs.

ICIs are the standard treatment for R/M HNSCC. However, ICIs show benefits in less than half of patients with R/M HNSCC, with a response rate of 25.8 to 39.4% [4,12]. Additionally, ICIs can cause deadly immune-related adverse events. Therefore, the selection of patients suitable for ICIs therapy is important. Unlike chimeric antigen receptor T-cell therapy, which uses genetically engineered T cells, ICI therapy utilizes the natural activity of the patient’s existing immune cells to target and kill cancer cells. Thus, its effectiveness depends on the baseline immune function of the patient. Therefore, patient status should affect ICI therapy outcomes. Previously reported patient factors prognostic of ICI therapy include age, sex [13], smoking status, performance status [14], and nutritional status. Among these, nutritional status has been shown to influence immune conditions through cytokine inhibition and immune cell production [15]. Therefore, the assessment of the nutritional status of patients receiving ICI therapy is imperative. Nutritional status is evaluated in various ways. Low body mass index (BMI), weight loss, and reduced muscle mass are used for the diagnosis of malnutrition [2] and are also established prognostic factors for HNSCC [16–18]. Additionally, blood markers such as serum albumin levels and composite scores such as the prognostic nutritional index (PNI) and geriatric nutritional risk index (GNRI) are also used to assess nutritional status. Among these parameters and indices, BMI has been the most commonly utilized indicator. However, BMI is not a prognostic factor in patients with HNSCC treated with ICIs [4,12]. In contrast, PNI, GNRI, and reduced muscle mass have been demonstrated to be associated with the survival of patients with HNSCC treated with ICIs [4,12]. Both PNI and GNRI are calculated using serum albumin levels. However, serum albumin is influenced not only by nutritional status but also by inflammation and hydration status, which may cause fluctuations and limit its reliability as a marker. In contrast, muscle mass is more stable over time, making it a more reliable indicator of nutritional status and a useful prognostic factor for patients undergoing ICI therapy.

Skeletal muscle mass assessed using CT has been identified as a prognostic factor of ICI therapy in various cancers [19]. Common assessment methods include the measurement of skeletal muscle mass at the L3 vertebral level, total muscle mass, and muscle density. The most widely used metric is the skeletal muscle index (SMI), which is calculated by dividing the skeletal muscle area at the L3 level by the square of the patient’s height. We previously demonstrated that SMI is an independent prognostic factor for both PFS and OS in patients with HNSCC treated with nivolumab [4]. However, in that study, approximately one-quarter of patients were excluded due to the absence of L3 level images. This limitation prompted us to explore alternative methods for assessing muscle mass, ultimately leading to the use of temporal muscle thickness as a substitute. Leitner et al examined the association between TMT and SMI in patients with brain metastases from melanoma or lung cancer [8]. The Spearman correlation coefficients were 0.65 in patients with lung cancer and 0.662 in those with melanoma, indicating a strong correlation between TMT and the SMI. Moreover, TMT has been shown to have significant prognostic value in patients with glioblastoma, central nervous system lymphoma, and brain metastases from lung cancer or melanoma [20]. In addition, TMT has been identified as an independent prognostic factor for PFS and OS in patients with HNSCC [9,10]. Thus, TMT has previously been investigated as an imaging-based surrogate marker of sarcopenia and as a prognostic factor in several malignancies. In this context, the present study extends previous findings by evaluating the association of TMT with objective response and survival outcomes specifically in patients with R/M HNSCC receiving ICIs. To our knowledge, this is among the first studies to examine TMT in this specific clinical setting. Our findings would be of practical value to oncologists, as TMT can be measured using routine imaging, providing a non-invasive method to assess patients’ physical status and prognostic risk during ICI therapy.

The additional sensitivity analyses supported the robustness of the association between TMT and survival outcomes. The association between TMT and PFS/OS was observed not only when TMT was dichotomized using sex-specific cutoffs, but also when TMT was analyzed as a continuous variable. Moreover, the association was not explained by BMI or apparent imbalance in treatment-related variables. Rather than representing an ICI-specific immune biomarker, TMT may reflect skeletal muscle reserve, physical status, and overall host condition. These host-related factors may influence clinical outcomes in patients receiving ICIs. Therefore, the present findings should be interpreted as demonstrating an association between TMT and clinical outcomes in an ICI-treated population, rather than establishing a mechanistically specific predictive biomarker of ICI efficacy.

Our study has several limitations. First, this is a retrospective study. Owing to the retrospective nature of the study, the interval between imaging and initiation of ICI therapy varied among patients. Although CT images obtained within 3 months before ICI initiation were used, temporal changes in muscle status during this interval may have affected the accuracy of TMT as a baseline marker. Furthermore, objective response was assessed retrospectively in routine clinical practice rather than according to a predefined study protocol. Future prospective studies with standardized imaging intervals and predefined response assessment protocols are warranted. Second, this study did not include a non-ICI control group. Therefore, the present findings should be interpreted as demonstrating the prognostic value of TMT in patients receiving ICIs, rather than establishing TMT as a treatment-specific predictive biomarker. Because all patients in this cohort received ICI therapy, we could not determine whether TMT specifically predicts benefit from ICIs or reflects general host-related prognosis. Third, the sample size of our cohort was relatively small. Consequently, not all potential covariates could be included in the logistic regression model, which may limit the robustness of the findings. Fourth, TMT was assessed using CT. In contrast, previous studies involving brain tumors used magnetic resonance imaging (MRI) for TMT evaluation [20]. MRI can provide superior soft tissue contrast, allowing for more accurate delineation of the temporal muscle. Therefore, the accuracy of TMT measurements in our study may have been suboptimal. Fifth, we did not directly assess immunological markers or treatment-induced immune responses. In addition, BMI was the only available nutrition-related variable in the present dataset; detailed nutritional and inflammatory markers, such as serum albumin, PNI, GNRI, weight-loss data, NLR, and CRP, were not available. Therefore, the relationship between TMT, nutritional status, systemic inflammation, and immune response warrants further investigation.

In conclusion, TMT is a practical and accessible imaging-based marker associated with clinical outcomes in patients with R/M HNSCC receiving ICIs. The present findings support the prognostic relevance of TMT for risk stratification in this population. However, because this study did not include a non-ICI control group, TMT should not be regarded as an established treatment-specific predictive biomarker at this stage. Several composite scoring systems incorporating both patient-related and tumor-related factors have been developed and validated for patients with HNSCC receiving ICIs [21]. Given its association with physical and prognostic status and survival outcomes, TMT may be a candidate host-related variable for future incorporation into such composite prognostic models. Further prospective studies are warranted to validate the utility of TMT and develop more robust models for prognostic assessment and treatment decision-making in patients receiving ICIs.

## Supporting information

S1 Data Anonymized dataset underlying the analyses. The dataset includes anonymized clinicopathological variables, temporal muscle thickness measurements, treatment information, response assessment, and survival outcomes used in this study.
(XLSX)

S1 Table Sensitivity analyses treating TMT as a continuous variable for PFS and OS. The table summarizes Cox proportional hazards models evaluating the association between mean TMT and survival outcomes. Mean TMT was analyzed as a continuous variable, and hazard ratios are presented per 1-mm increase in mean TMT.
(XLSX)
